# Low nitrogen retention in a Japanese cedar plantation in a suburban area, western Japan

**DOI:** 10.1038/s41598-021-84753-1

**Published:** 2021-03-05

**Authors:** Ru Yang, Masaaki Chiwa

**Affiliations:** 1 Graduate School of Bioresource and Bioenvironmental Sciences, Kyushu University, Fukuoka, Japan; 2 Kyushu University Forest, Kyushu University, 394 Tsubakuro, Sasaguri, Fukuoka, 811-2415 Japan

**Keywords:** Biogeochemistry, Element cycles

## Abstract

This study aimed to evaluate nitrogen (N) leaching from Japanese cedar, the main plantation species in Japan, in response to elevated atmospheric N deposition. N leaching and possible factors, including soil nitrification, tree N uptake, and topographic steepness, were evaluated in mature (64–69 year) Japanese cedar trees planted on steep slopes (25°–40°) and neighboring Japanese oak plantations in suburban forests, which served as reference sites. N fertilization (50 kg N ha^−1^ year^−1^ as ammonium nitrate) was conducted to evaluate the response of N leaching to an elevated inorganic N pool in the surface soil. The soil water nitrate (NO_3_^−^) concentration below the rooting zone in the Japanese cedar forest (607 ± 59 μmol L^−1^) was much higher than that in the Japanese oak plantations (8.7 ± 8.1 μmol L^−1^) and increased immediately after fertilization, indicating high N leaching from the Japanese cedar plantations. The relatively low N uptake by Japanese cedar planted on the steep slopes could be an important contributor to the high N leaching. This study highlights the importance of vegetation composition for managing the water quality in headwater streams from forest ecosystems disturbed by atmospheric N deposition.

Human activities, such as fuel combustion and agricultural activities, have increased anthropogenic nitrogen (N) deposition, especially since 1960^1^. Chronic high N deposition has strongly influenced N cycling and may potentially cause N saturation in forested ecosystems, leading to high levels of N leaching from these ecosystems^2^. Extensive studies in Europe^3^, China^4^, North America^5^, and Japan^6,7^ have shown the major role of atmospheric deposition on elevated nitrate (NO_3_^−^) concentrations in stream water from forest ecosystems, contributing to downstream eutrophication^8,9^.

Although the effects of elevated atmospheric N deposition on N leaching from forested ecosystems are widely recognized, there are various responses of N leaching to elevated atmospheric N deposition among forested watersheds. Dise and Wright^3^ showed that the response of N leaching is variable among forested watersheds with atmospheric N deposition from 10 to 25 kg N ha^−1^ year^−1^ throughout Europe. This is because there are many factors that affect the response of NO_3_^−^ leaching from forested watersheds to atmospheric N deposition^10–12^, including successional age^13–17^, vegetation and soil characteristics^11,18–21^, and topography^22^. Understanding the factors affecting N leaching from forested watersheds is critical for managing the water quality in headwater streams.

In Japan, several studies have investigated the factors affecting the responses of N leaching to elevated N deposition. Many studies^10,23–26^ have shown that high NO_3_^−^ concentrations in stream waters are observed near urban or suburban forested watersheds, where atmospheric N deposition is high. It has also been suggested that there is significant N leaching from the main planting areas for Japanese cedars^24–26^. Because Japanese cedar plantations account for approximately 44% of the plantation area and 18% of the total forested area in Japan, N leaching from Japanese cedar plantations should be intensively evaluated. However, N leaching from Japanese cedar plantations is not completely understood.

Many studies have evaluated N leaching from Japanese cedar plantations indirectly by statistical analysis in which synoptic sampling of stream water was conducted within a forested watershed containing various overstory species^25,26^. Stand-scale investigations can directly evaluate N leaching from specific vegetation types^27–29^. However, studies evaluating N leaching from Japanese cedar plantations at the stand scale are limited to studies in suburban forests in eastern Japan by Oyanagi et al.^30^.

The presence of arbuscular mycorrhizae (AM)^31^ in Japanese cedar forests could lead to high NO_3_^−^ leaching from plantations of this species. The high chemical quality of needle litter in AM-dominated stands results in rapid C mineralization, therefore contributing to a relatively low soil C/N ratio in AM-associated forests^32^. Phillips et al.^32^ revealed that there is an inorganic nutrient economy in AM-dominated forests, owing to the lower soil C/N ratio, which increases the risk of NO_3_^−^ leaching because of the high rate of nitrification^28^. Midgley and Phillips^33^ indicated that forests with AM leach more NO_3_^−^ in response to elevated atmospheric N deposition.

In addition, high NO_3_^−^ leaching should be evaluated from a forestry point of view. In Japan, Japanese cedar was extensively planted in the 1950s and 1960s, and these trees are now reaching maturity (> 50 years). Fukushima et al.^34^ revealed that the amount of N uptake by Japanese cedars decreases as the trees age. Therefore, N retention, defined as biological utilization (including tree N uptake), might also increase the likelihood of N leaching from mature Japanese cedar plantations in response to elevated N deposition. Topography also has a large influence on stream N leaching. NO_3_^−^ leaching largely occurs in relatively steep catchments^26,35^. Because many Japanese cedars are planted on steep slopes in mountainous areas in Japan^36^, steep topography might also be a reason for the high NO_3_^−^ leaching in plantations of this species.

The objectives of this study are (1) to evaluate stand-scale N leaching and retention in Japanese cedar plantations in suburban forests in western Japan and (2) to explore possible factors affecting N retention in a Japanese cedar plantation.

## Results and discussion

### High N leaching and low N retention in a suburban Japanese cedar plantation

**Figure 1:**
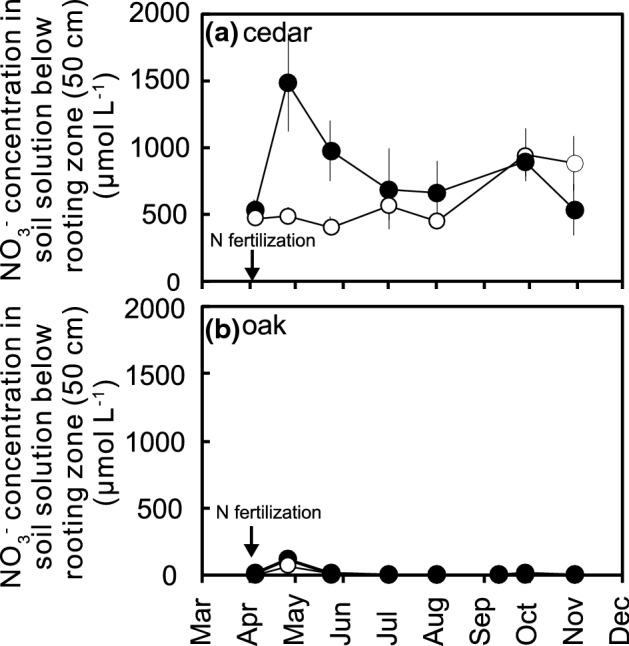
Changes in the NO_3_^−^ concentration in the soil solution below the rooting zone (50 cm depth) in the (**a**) Japanese cedar and (**b**) oak plantations during the study period from April to November 2018. The error bars indicate the standard errors (*n* = 5). The open circles refer to the unfertilized plots; the closed circles refer to the fertilized plots. The arrows indicate the timing of the addition of N fertilizer (50 kg N ha^−1^ year^−1^ as ammonium nitrate) to the surface of the forest floor. A two-way repeated measures ANOVA indicated that the difference is significant between tree species (cedar and oak) (*p* < 0.01), fertilization (*p* < 0.05), and their interactions (*p* < 0.05).

The NO_3_^−^ concentration in the soil solution below the rooting zone (collected from a depth of 50 cm) in the unfertilized plots of the Japanese cedar plantation (607 ± 59 μmol L^−1^) was much higher than that in the unfertilized plots of the Japanese oak plantation (8.3 ± 8.1 μmol L^−1^) (Table 1). In addition, the difference between the two sites was consistent during the observation period from April to November 2018 (Fig. 1). Higher NO_3_^−^ concentration in the Japanese cedar plantation than in the Japanese oak plantation indicates high levels of NO_3_^−^ leaching from the Japanese cedar plantation. The high NO_3_^−^ leaching from the Japanese cedar plantations in this study was consistent with the findings of a study by Oyanagi et al.^30^, which showed that the NO_3_^−^ concentration in a soil solution from deeper soil (50 and 80 cm depths) was high (approximately 240 μmol L^−1^) in a N-saturated Japanese cedar plantation in eastern Japan, where high levels of atmospheric N deposition (10.5 kg N ha^−1^ year^−1^) and stream water N leaching (13.5 kg N ha^−1^ year^−1^) have been observed^7^.

**Table 1:** Average NO_3_^−^ concentration in soil solution below rooting zone (50 cm depth), soil chemical properties of A layer (0–5 cm) in unfertilized plots of Japanese cedar and oak plantations during the study period from April to November 2018.

	Japanese cedar	Japanese oak	*p* value
NO_3_^−^ concentration in soil solution (μmol L^−1^)	607 ± 59^a^	8.7 ± 8.1	***^c^
Total C (%)	6.38 ± 1.3	5.78 ± 0.35	ns^d^
Total N (%)	0.37 ± 0.06	0.35 ± 0.06	ns^d^
C/N ratio	17.0 ± 1.1	16.2 ± 0.8	ns^d^
Inorganic N (mg N kg^−1^)	9.81 ± 1.24	7.07 ± 0.68	ns^d^
Net mineralization (mg N kg^−1^ day^−1^)	0.71 ± 0.38	0.71 ± 0.26	ns^d^
Net nitrification (mg N kg^−1^ day^−1^)	0.63 ± 0.36	0.67 ± 0.26	ns^d^
Relative net nitrification^b^ (%)	90 ± 13	96 ± 4	ns^d^

^a^Mean ± standard error (*n* = 5).

^b^Defined as the proportion of net nitrification to net N mineralization.

^c^Significant difference (*p* < 0.001 for *t*-test).

^d^No significant difference (*p* > 0.05 for *t*-test).

Watanabe et al.^26^ reported that catchments with high coniferous coverage, including Japanese cedar plantations near urban areas, tend to have high NO_3_^−^ concentrations in stream water. It has also been reported that there is significant N leaching from forested watersheds in which the main species is Japanese cedar^24–26^. However, N leaching from Japanese cedar plantations has been evaluated indirectly by statistical analysis in which synoptic sampling of stream water was conducted within a forested watershed containing various overstory species^25,26^. Our stand-scale investigation confirmed the presence of high N leaching from Japanese cedar plantations in suburban areas.

The NO_3_^−^ concentration in the soil solution collected from a depth of 50 cm in the fertilized plots of the Japanese cedar plantation increased immediately after fertilization and then returned to the same NO_3_^−^ concentrations found in the unfertilized plots (Fig. 1a). In contrast, there was only a small increase in NO_3_^−^ concentration in the fertilized plots of the Japanese oak plantation after fertilization (Fig. 1b). Another experiment also showed that the response of NO_3_^−^ concentration in the soil solution from below the rooting zone is variable in response to N addition and that the response of N leaching is faster in coniferous forests than in deciduous forests in stand-scale fertilization experiments^37^. The immediate NO_3_^−^ leaching in the Japanese cedar plantation and the small increase in leaching in the Japanese oak plantation observed in this study after fertilization suggest that the response of N leaching to N addition is variable.

The NO_3_^−^ concentration in the soil solution in the Japanese cedar plantation increased approximately three-fold after the addition of ammonium nitrate at a rate of 50 kg N ha^−1^ (Fig. 1a). Considering the current atmospheric N deposition in this study area^38^ (approximately 16 kg N ha^−1^ year^−1^), it is likely that NO_3_^−^ leaching in Japanese cedar plantations has increased in response to elevated atmospheric N deposition.

### Possible mechanisms for high N leaching and low N retention in Japanese cedar plantations

A high soil nitrification rate may increase the risk of NO_3_^−^ leaching^28^. Because Japanese cedar is an AM-associated species^31^ that is likely to leach more NO_3_^−^ in response to N deposition^33^, high N leaching from Japanese cedar plantations may be caused by high soil net nitrification rates. The soil net mineralization rates in the unfertilized Japanese cedar plots in this study (0.71 ± 0.38 mg N kg^−1^ day^−1^; Table 1) were comparable to those (0.9 mg N ka^−1^ day^−1^) in the neighboring forests, where high N leaching via stream water has been reported^23^. The lower end of the range of soil nitrification rates (0.63 ± 0.36 mg N kg^−1^ day^−1^; Table 1) were also similar to those in Japanese cedar plantations in southern Japan (0.29 mg N kg^−1^ day^−1^)^39^ and central Japan (0.3 mg N kg^−1^ day^−1^)^34^. In addition, the relative net nitrification, defined as the proportion of net nitrification to net N mineralization, was 90% in the Japanese cedar plantation (Table 1). These results indicate that soil nitrification in the Japanese cedar plantation in this study was high enough to cause significant N leaching.

However, the soil net nitrification in the Japanese cedar plantation was not significantly higher than that in the Japanese oak plantation (Table 1). The lack of significant differences in the soil total C, total N, C/N ratio, and inorganic N between the Japanese cedar and oak plantations (Table 1) may explain the lack of significant differences in the soil net nitrification between the plantations, because soil N status generally affects soil nitrification rates^28^. In addition, NO_3_^−^ leaching was not correlated with soil net nitrification (Table 2). Therefore, soil nitrification could not explain the difference in NO_3_^−^ leaching from the Japanese cedar and oak plantations, although soil nitrification may be an important source of NO_3_^−^ leaching in Japanese cedar plantations.

**Table 2:** Coefficient of determination and regression coefficients of multiple linear regression of NO_3_^−^ leaching.

Variable	NO_3_^−^ leaching (μmol L^−1^)	*p*
R^2^	0.874	< 0.001
Intercept	913	< 0.001
Nitrification (mg N kg^−1^ day^−1^)	–	0.700
N uptake (kg N ha^−1^ year^−1^)	− 22.9 (− 0.935)	< 0.001
Slope (°)	–	0.686

Numbers in parentheses indicate standardized coefficients. Stepwise *F* < 0.05.

The finding that soil nitrification could not explain the difference in NO_3_^−^ leaching between the Japanese cedar and oak plantations is consistent with the results of the N fertilization experiments in this study. N fertilization enhances the contents of inorganic N in surface soil. The inorganic N contents in surface (5 cm) soil after the N fertilization were calculated to be 303 and 190 mg N kg^−1^ for Japanese cedar plantation and Japanese oak plantation, respectively, which was higher than those before the N fertilization (Table 1). The smaller increases in NO_3_^−^ leaching despite the elevated inorganic N contents caused by N fertilization in the Japanese oak plantations than in the Japanese cedar plantation in this study (Fig. 1) suggest that other factors that lower N retention and subsequently increase N leaching are also important for NO_3_^−^ leaching.

Generally, N uptake by trees is an important factor regulating N retention^17,24^. N uptake was significantly lower in the Japanese cedar plantation than in the oak plantation (Table 3). The low N uptake in the Japanese cedar plantation compared with that in the Japanese oak plantation is supported by the lower N biomass pool and soil N storage in the Japanese cedar compared with the Japanese oak (Supplementary Table S1), even though the Japanese cedar is older than the Japanese oak (Supplementary Table S2). In addition, NO_3_^−^ leaching was significantly correlated with tree N uptake (Table 2), indicating that the lower N uptake by the Japanese cedars could be an important reason for the high N leaching in plantations of this species. The low N uptake in the Japanese cedar plantation compared to that in the Japanese oak plantation may also be supported by other studies showing that broadleaf forests have significantly higher N uptake than coniferous forests in China^40^ and Europe^41^. In addition, it has been shown that Japanese cedar is characterized by lower N uptake than other species, including deciduous hardwood and Japanese cypress forests^42^.

**Table 3:** Estimates of N uptake by Japanese cedar and Japanese oak plantations.

	N concentration of leaf fall (A) (%)	weight of leaf fall (B) (g m^−2^)	N uptake (A × B) (kg N ha^−1^ year^−1^)
Japanese cedar	0.62 ± 0.04^a^	217 ± 18	13 ± 1.1
Japanese oak	0.91 ± 0.03**^,b^	433 ± 33**	39 ± 3.0**

^a^Mean ± standard deviation (*n* = 5).

^b^Significant difference (*p* < 0.01 for *t*-test).

In this study, N uptake, calculated by multiplying the leaf fall amount by the N concentration in the leaf fall, may be underestimated because the annual increase in the N stored in the woody biomass was not included. However, the finding that the Japanese cedar plantation had a lower N uptake than the Japanese oak plantation is reliable because the annual N increase in the woody biomass in the mature (64–69 year) Japanese cedar plantations used in this study would be smaller than that in the Japanese oak plantations because woody N storage is lower in the Japanese cedar plantation than in the Japanese oak plantation (Supplementary Table S1), even though the Japanese cedar is older than the Japanese oak at this study site (Supplementary Table S2).

N uptake by trees is also affected by tree age^7^. Fukushima et al.^34^ evaluated tree N uptake at different ages (5–89 years) and demonstrated that N uptake by Japanese cedar was higher in younger stands (16 years old: 53 kg N ha^−1^ year^−1^) than in older stands (31 years old: 29 kg N ha^−1^ year^−1^, 42 years old: 24 kg N ha^−1^ year^−1^, and 89 years old: 34 kg N ha^−1^ year^−1^). The Japanese cedar plantation in this study is 64–69 years old and has reached maturity, resulting in high susceptibility to NO_3_^−^ leaching in response to elevated N deposition because of the reduction in tree N uptake with age. Therefore, low N uptake owing to forest maturity could be an important factor causing the low N retention in the Japanese cedar plantations.

The steep slope in the Japanese cedar plantation could be an additional reason for the low N retention. However, NO_3_^−^ leaching was not correlated with slope in this study (Table 2). Generally, stream NO_3_^−^ concentrations tend to be higher on steep slopes^26,35^. Steep slopes increase N flushing through water discharge^22,35^, as NO_3_^−^ leaching occurs from upper soils following stormflows. Because N leaching loss was significantly higher in Japanese cedar planted on steep slopes than in Japanese oak, a steep slope may also support high leaching losses in the cedar plantation. A greater range of slope conditions will be required in future research to examine the topographic effect on NO_3_^−^ leaching. It is possible that other factors, including soil microbial immobilization, lowered N retention. Soil microbial NO_3_^−^ immobilization is reportedly lower in Japanese cedar plantations than in broadleaf and Japanese cypress forests^43^. Further investigations are required to explore the mechanisms lowering N retention in Japanese cedar plantations.

## Conclusions

This study conducted a stand-scale investigation of N leaching from a specific species. The results confirmed high NO_3_^−^ leaching from a Japanese cedar plantation. N fertilization experiments indicate that the high NO_3_^−^ leaching observed in the Japanese cedar plantation could have been caused by low N retention. Although soil nitrification in Japanese cedar plantations could be an important source of NO_3_^−^ leaching, soil nitrification was not correlated with NO_3_^−^ leaching. The relatively low N uptake by Japanese cedar planted on the steep slopes could be an important contributor to the high N leaching. Because Japanese cedar is the main plantation species, accounting for approximately 44% of the plantation area in Japan, N leaching from these plantations may be an important source of N in headwater streams. Further investigations are required to explore the reasons for low N retention in Japanese cedar plantations.

## Materials and methods

### Study site

This study was conducted in two forest stands, a Japanese cedar (*Cryptomeria japonica*) plantation and a neighboring Japanese oak (*Quercus crispula*) plantation, both located in the Kasuya Research Forest (KRF; 33° 38′ N, 130° 33′ E) of Kyushu University, situated 15 km west of the Fukuoka metropolitan area (Supplementary Fig. S1). In this region, high levels of NO_3_^−^ leaching from headwater streams are observed^8,9^. The mean annual temperature and precipitation are 15.9 °C and 1769 mm over the last 10 years, respectively^38^. The atmospheric N deposition via bulk and throughfall and stemflow deposition in the Kasuya Research Forest are 11.3 and 15.5 kg N ha^−1^ year^−1^, respectively^38^. We compared plantations of Japanese cedar, the main plantation species in Japan, and Japanese oak, the dominant deciduous species across Japan, which served as a reference for evaluating the impact of tree species on N leaching. The distance between the two stands is approximately 3 km. Previously, these sites had been under natural forest and were planted for Japanese cedar in 1949–1954 and Japanese oak in 1994. Topography, tree age, diameter at breast height (DBH), and tree density in the Japanese cedar and oak plantations are shown in Supplementary Table S2.

In the Japanese cedar plantation, five 10 m × 10 m and five 15 m × 15 m plots with 2.5 m treated buffers on all sides (10 plots in total) were established as unfertilized and fertilized plots, respectively. In the Japanese oak forest plantation, one 10 m × 10 m plot and one 15 m × 15 m plot with 2.5 m treated buffers on all sides (in total 2 plots) were established as a unfertilized and a fertilized plot, respectively. In the fertilized plots, N fertilization (50 kg N ha^−1^ year^−1^ as ammonium nitrate) was conducted at the beginning of April 2018. The purpose of the fertilization treatment in this study was to evaluate the short-term response of N leaching to an elevated inorganic N pool in the surface soil in Japanese cedar and oak plantations. N fertilizer was added directly to the forest floor.

### Sample collection

Soil solutions were collected monthly from April to November 2018 using a tension lysimeter with a porous cup to evaluate N leaching. Each lysimeter was installed at a depth of 50 cm below the predominant rooting zone so that the fraction of soil solution that represents potential leachate could be obtained. Above the surface, we used an injection syringe to collect the soil solution; the syringe was set up on the first day, and we collected the soil solution the next day. After collection, the soil solution samples were transported to a laboratory within approximately 4 h and immediately filtered by passing through a prewashed 0.45-μm membrane filter (Pall, Ekicrodisc syringe filter). Then, the concentration of NO_3_^−^ was analyzed by ion chromatography (Aquion, Dionex, Japan). Because the NH_4_^+^ content in the soil solution was negligible, the data are not shown in this paper.

The soil net N mineralization and nitrification in the surface mineral soil (0–5 cm) in the unfertilized plots of the Japanese cedar and oak plantations were measured using in situ incubation of buried bags following the methods detailed in Eno^44^. The collected soils were divided into two subsamples. The first was used to determine the initial mineral N content and the second was buried in the field at the same time and same depth for the incubation (0–5 cm). Soil incubation was conducted from 31st March to 30th June 2018 and from 30th June to 31st October 2018. The soils were sieved through a 4 mm screen before incubation, and roots and coarse organic matter were discarded. The soils were extracted at the beginning and end of the incubation period using 2 N KCl. The extracted solution was analyzed for NH_4_^+^ by the indophenol blue method, and NO_3_^−^ was determined spectrophotometrically after cadmium reduction. The net mineralization and nitrification rates were calculated as the difference in the NH_4_^+^ and NO_3_^−^ pools before and after in situ incubation, respectively, by quantifying the changes in NH_4_^+^ and NO_3_^−^ over the same time period.

The soil samples used for the soil C and N analyses were also collected in the surface mineral soil (0–5 cm). They were passed through a 2 mm sieve and air-dried, and the sieved dry soil samples were gently ground with a mortar and pestle. The C and N concentrations were measured by the combustion method (MT-700, Yanaco, Kyoto, Japan).

To evaluate the uptake of N and its effect on N retention by plants, litterfall was collected from the Japanese cedar plots and oak control plots from April 2018 to March 2019. N uptake was evaluated as the N amount contained in the leaf fall. The N amount in the leaf fall was calculated by multiplying the leaf fall weight by the N concentration in the leaf fall. One and 5 litter traps (0.5 m^2^ in size) per plot were established in the Japanese cedar and oak stands, respectively. Each litter trap was constructed from a steel hoop (80 cm aperture) and a woven nylon bag (80 cm depth) and was positioned 1 m above the ground with plastic stakes. Samples were dried for 48 h at 80 °C, sorted into leaf fall and other material, and weighed. The N concentrations were measured by the combustion method (CN Corder MT-700, Yanaco, Kyoto, Japan).

The woody and leaf N biomass were calculated to evaluate the biomass N pool. Woody N biomass was calculated by multiplying the wood mass by the trunk N concentration. The wood mass (kg) was determined using the following allometric equation^43^:

$$ W\, = \,0.1853\rho \, DBH^{2.491} , $$

where DBH is the diameter at breast height (cm) and *ρ* is the specific weight (kg m^−3^) for cedar (0.352) and oak (0.523)^43^. The trunk N concentration used for the calculation was 0.12% for Japanese cedar^45^ and 0.39% for Japanese oak^46^. The leaf N biomass was calculated by multiplying the leaf mass by the leaf N concentration. The leaf mass was determined from the leaf lifetime and weight of leaf fall collected from the Japanese cedar and Japanese oak plots from April 2018 to March 2019. The leaf N concentration used for the calculations was 1.06% for Japanese cedar^45^ and 1.80% for Japanese oak, as determined in this study. Surface (0–5 cm) soil N storage was calculated by multiplying total N concentration by soil density (0.33 g cm^−3^ for Japanese cedar and 0.55 g cm^−3^ for Japanese oak).

### Statistics

The effects of tree species (Japanese cedar and Japanese oak), fertilization, and their interactions on NO_3_^−^ leaching were analyzed using two-way repeated measures ANOVA. A linear regression model that can evaluate the relationship between a scalar response and multiple explanatory variables was used to evaluate the multiple factors (nitrification, N uptake, and slope) influencing NO_3_^−^ leaching. The coefficient of determination for the regression model was 0.874 at a significance level of *p* < 0.001.

$$ NO_{3}^{-} leaching = a_{0} + \, a_{{1}} Nit \, + \, a_{{2}} Nup + a_{{3}} Sl, $$

where *Nit*, *Nup*, and *Sl* are soil net nitrification (mg N kg^−1^ day^−1^), tree N uptake (kg N ha^−1^ year^−1^), and slope (°), respectively. Comparisons of soil chemical properties and N uptake between Japanese cedar and oak plantations were performed using *t*-tests. All the statistical analyses were performed using SPSS 22.0J (SPSS Japan Inc, Tokyo, Japan).

## Supplementary Information

Supplementary Information.
