# Supplementary material for: Low nitrogen retention in a Japanese cedar plantation in a suburban area, western Japan
Source: Sci Rep. 2021 Mar 5;11:5335. doi: 10.1038/s41598-021-84753-1 (PMC7935847; doi:10.1038/s41598-021-84753-1)
Supplement: Supplementary file 1 — Supplementary Information. [file 41598_2021_84753_MOESM1_ESM.docx]

**Supplementary material**

Low nitrogen retention in a Japanese cedar plantation in a suburban area, western Japan

Ru Yang^1^, Masaaki Chiwa^2*^

^1^ Graduate School of Bioresource and Bioenvironmental Sciences, Kyushu University;

^2^ Kyushu University Forest, Kyushu University, 394 Tsubakuro, Sasaguri, Fukuoka, 811-2415, Japan

*Corresponding author: mchiwa@forest.kyushu-u.ac.jp

The SI contains one figure and two tables.

**Figure S1.** Location of a) the study site (KRF; Kasuya Research Forest) and b) experimental stands in the Japanese cedar and oak plantations. The map was generated from Google Maps.

| Table S1. Estimates of N biomass and soil (0-5 cm) N storage in Japanese cedar and Japanese oak plantations. | | | | | |
| --- | --- | --- | --- | --- | --- |
|  | Aboveground N biomass (kg N ha^–1^) | | | Soil (0-5 cm) N storage (kg N ha^–1^) |  |
|  | Wood | Leaf | Sum |  |  |
| Japanese cedar | 524 | 69 | 593 | 610 |  |
| Japanese oak | 1132 | 78 | 1210 | 921 |  |
|  | | | | | |

| Table S2. Topographic slope, age of tree, diameter of breast height (DBH), density in Japanese cedar and Japanese oak plantations. | | | | |
| --- | --- | --- | --- | --- |
|  | Slope (°) | Age of tree (year) | DBH (cm) | Tree density (trees ha^–1^) |
| Japanese cedar | 25–40 | 64-69 | 33.0 ± 0.9 ^a^ | 1040 |
| Japanese oak | 3 | 24 | 22.8 ± 1.5 | 1600 |
| ^a^ mean ± standard deviation (*n* = 5) | | | | |
